# The Incidence of Stroke Mimics in the Emergency Department of a Tertiary-care Center in Lebanon

**DOI:** 10.5811/westjem.39718

**Published:** 2025-07-18

**Authors:** Hind Anan, Maya Bizri, Mustapha Jomaa, Nour Ibrahim, Afif Mufarrij

**Affiliations:** * American University of Beirut Medical Center, Department of Emergency Medicine, Beirut, Lebanon; † Cleveland Clinic Foundation, Neurologic Institute, Department of Psychiatry and Psychology, Cleveland, Ohio; ‡ American University of Beirut Medical Center, Department of Psychiatry, Beirut, Lebanon

## Abstract

**Introduction:**

Stroke mimics comprise a significant proportion of cases presenting with neurological deficits and can be difficult to differentiate from true stroke cases. Our aim in this study was to assess the frequency and etiologies of stroke mimics presenting to our emergency department (ED).

**Methods:**

We conducted a retrospective review of the charts of patients presenting to the ED of a tertiary-care center between November 2018–August 2023 and on whom the stroke code was activated. The cases were categorized into real strokes or stroke mimics based on patients’ discharge diagnoses.

**Results:**

Stroke code activation was implemented on 584 patients during the study period. These patients received full service and a final discharge diagnosis. Of these, 349 (59.8%) received a diagnosis of a true stroke, whether ischemic, hemorrhagic, or transient ischemic attack. The remaining 235 (40.2%) were classified as stroke mimics, with functional (12.8%) and medical (87.2%) etiologies. Medical stroke mimics were further categorized into non-cerebrovascular neurologic (59.5%), infection or allergic reaction (17.1%), cardiovascular (11.7%), metabolic or drug-induced (8.3%), and other (3.4%). Factors found to favor stroke mimics were history of neurological (adjusted odds ratio [aOR] 4.98; 95% confidence interval [CI] 2.89 – 8.57) or psychiatric disorders (aOR 2.88; 95% CI 1.29 – 6.41) and patients presenting with altered mental status (aOR 1.70; 95% CI 1.04 – 2.80) or generalized weakness (aOR 2.38; 95% CI1.12 – 5.03). Conversely, factors that favored true strokes (with OR <1 for mimics), were patients aged >65 years (aOR 0.61; 95% CI 0.38–0.96), history of hypertension (aOR 0.61; 95% CI 0.38 – 0.97) or atrial fibrillation (aOR 0.39; 95% CI 0.21 – 0.72), and presenting with speech disturbance (aOR 0.56; 95% CI 0.37–0.83) or extremity weakness (aOR: 0.22; 95% CI 0.15–0.38).

**Conclusion:**

Approximately 40% of cases presenting to our ED with stroke code activation were found to be mimics. The high ratio warrants the establishment and adoption of a more specific triaging algorithm for stroke code activation to minimize the pressure on an already overburdened healthcare sector.

## INTRODUCTION

In 2019, stroke was the second leading cause of death globally, accounting for 11.6% of deaths annually.^1^ Given the high rate of mortality and disability, it is essential to investigate and treat any presenting stroke patient in a timely manner. This requires a host of diagnostic tests and, possibly, subsequent thrombolytic treatment. The costs add up to further stress an already burdened healthcare system. Globally, there is one new stroke every three seconds, leading to a worldwide cost exceeding 1% of the global gross domestic product.^2^ This high burden makes it crucial to distinguish a stroke from any similar mimic. Stroke mimics (SM) are defined as stroke-like symptoms and presentations that arise due to a non-cerebrovascular etiology.^3^ Such mimics are estimated to make up an average of 22% of all stroke presentations, ranging from 1–64% of all suspected stroke cases.^4^

Stroke mimics have a wide range of etiologies, with seizures, migraines, and functional disorders making up more than 40% of the cases.5,6 Other causes include cerebrovascular narrowing, toxic or metabolic origins, brain trauma and subdural hematoma, infection, and cardiovascular and other neurologic disorders.5 Patients experiencing stroke mimics exhibit different characteristics from those experiencing an actual stroke. These include differences in age, gender, comorbidities, and presenting signs and symptoms.^7^–^14^

An improper identification of an SM can subject a patient to a myriad of diagnostic tests and imaging, and potentially to an unnecessary thrombolytic treatment. It is estimated that between 1–16% of SM patients receive thrombolysis with tissue plasminogen activator (tPA), with 0.5% of these patients developing a symptomatic intracerebral hemorrhage.^15^ Such unnecessary interventions can add up, with an estimated cost of treatment of $5,400 per admission.^16^ These factors necessitate the early identification of an SM patient to spare the unwarranted tests and interventions.

Stroke is also a leading cause of mortality in Lebanon, with 3.1% of total deaths in 2021 attributed to cerebrovascular incidents.17 The prevalence of stroke was estimated to be 0.5%^18^, with an average cost of US $6,961 per stroke patient.19 Moreover, a survey of Lebanese people above the age of 40 indicated that 12.1% had experienced at least one stroke symptom.20 Data on the incidence of SMs in Lebanon is scarce. This study aimed to assess the incidence of stroke presentations to a local emergency department (ED) and explore the subsequent diagnoses related to SMs. We looked at patient characteristics, tests and interventions performed, and final diagnoses upon discharge.

## METHODS

### Study Design and Sample

This was a retrospective, descriptive study of adult patients presenting to the ED of a tertiary-care center in Lebanon. We reviewed the records of all adult patients aged ≥18 years on whom the stroke code was activated upon presentation to our ED between November 2018–August 2023. In our facility, the stroke code is typically activated after the initial assessment of the patient upon arrival. The activation is triggered either directly by the senior attending in the ED or after confirmation with them.

The study was approved by our institutional review board (BIO-2020-0293) and followed the STROBE (Strengthening the Reporting of Observational Studies in Epidemiology) statement.

**Population Health Research Capsule**

What do we already know about this issue?

*A significant proportion of individuals presenting with stroke-like symptoms are ultimately diagnosed with a stroke mimic rather than true stroke*.

What was the research question?

*What are the frequency and etiologies of stroke mimics presenting to our tertiary-care center emergency department?*

What was the major finding of the study?

*Among 584 patients, 235 (40.2%) received a diagnosis of stroke mimic with functional (12.8%) or medical (87.2%) etiologies*.

How does this improve population health?

*The study highlights the need for a more specific screening pathway for stroke-code activation to reduce strain on an already burdened healthcare system*.

### Data Collection and Variables Measured

Patients were identified using the administrative data of all stroke code activations during the study period. Patients who left against medical advice or were transferred to another hospital before a diagnosis could be made were excluded from the analysis. All study data were extracted manually from the electronic health record (EHR) (Epic Systems Corporation, Verona, WI). Data abstractors, who had prior training in using the EHR for clinical purposes, were not blinded to the study hypothesis.

The data collection protocol was standardized, with variables defined prior to the analysis. Collected variables included patient demographics and characteristics, vital signs upon presentation to the ED, presenting symptoms, imaging studies performed, thrombolysis interventions given, disposition, and discharge diagnoses. We reported missing variables as “unknown.”

### Diagnoses

We used the *International Classification of Diseases, 10^th^ Rev*, Clinical Modification (ICD-10-CM) coded diagnoses to classify patients into nine categories: ischemic stroke; hemorrhagic stroke; transient ischemic attack (TIA); functional disorders; neurological disorders other than cerebrovascular accidents; infections or allergic reactions; cardiovascular; metabolic or drug-induced; and diagnoses not elsewhere classified. True strokes were defined as TIA, ischemic stroke, or hemorrhagic stroke confirmed on brain imaging. The six remaining categories were classified under SMs.

### Medical Record Review Studies Criteria

This study followed recommended practices for retrospective chart reviews, as described by Worster et al.^21^ Details of individual method criteria followed are described in Table 1.

### Statistical Analysis

We performed statistical analysis using SPSS Statistics v28.0 (IBM Corp, Armonk, NY). Significance was set at an alpha of 0.05. We presented categorical variables as percentages and frequencies, while continuous variables were expressed as means ± standard deviation or median and interquartile range (IQR). We used chi-squared and Fisher’s exact tests to compare groups of categorical variables, and the *t*-test and Mann-Whitney U tests to compare the differences in numerical variables. A multivariable logistic regression model was constructed to determine independent factors associated with SMs. Variables were included in the model if they were clinically relevant or found to be significant on bivariate analysis.

## RESULTS

Over a 58-month period, 654 patients presented to our ED and had a stroke code activation. Of these, 70 (10.7%) patients either left against medical advice or were transferred to another facility after being deemed stable and having received incomplete service. Patients who did not have a definitive diagnosis were excluded from the analysis. Of the 584 patients who had a known diagnosis at discharge, 349 (59.8%) had a true stroke, 249 (71.3%) were diagnosed with ischemic stroke, 37 (10.6%) with hemorrhagic stroke, and 63 (18.1%) with TIA.

**Figure 1:**
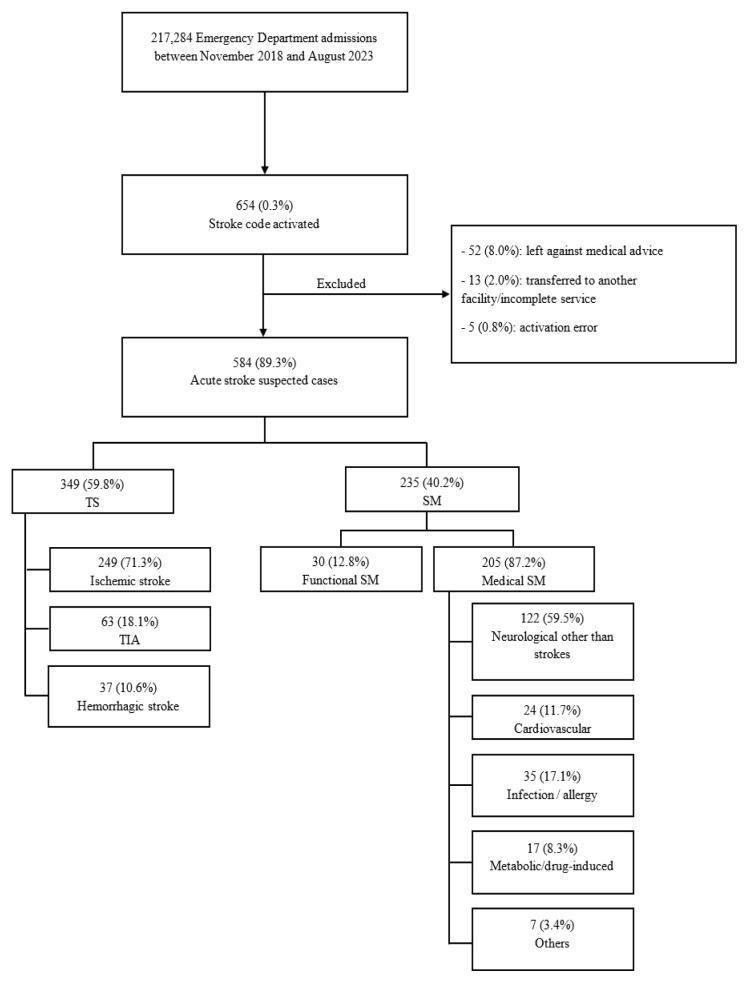
Flow diagram of patient selection from all stroke code activations between November 2018–August 2023: diagnostic classification into true stroke and stroke mimic. *TS*, true stroke; *SM*, stroke mimic; *TIA*, transient ischemic attack.

The remaining 235 (40.2%) were diagnosed with a stroke mimic. Of those, 30 (12.8%) had a functional SM (ie, a psychiatric etiology of their symptoms), while 205 (87.2%) had a medical SM (non-cerebrovascular, non-psychiatric origin of symptoms. Neurological disorders other than strokes were the most common presentation (59.5%) in the medical SM, and seizures were the most common presentation, comprising 27.0% of neurological SM and 14.0% of all SM (Figure 1).

Table 2 shows the demographics and ED visit characteristics of the identified patients. Stroke patients were significantly older than stroke-mimic patients (70.6 [±13.5] years vs 64.7 [±16.0], respectively; *P* = .001). There was no significant difference in gender, marital, or smoking status between the two groups. Stroke patients were more likely to have hypertension (72.5% vs 61.3%; *P* = .004), coronary artery disease (31.8% vs 18.7%; *P* = .001), atrial fibrillation (23.5% vs 8.5%; *P* < .001), and previous ischemic stroke (21.0% vs 10.6%; *P* = .001). On the other hand, a history of psychiatric (11.5% vs 4.3%; *P* = .001) and neurological disorders (29.8% vs 9.7%; *P* < .001) was more common in SM patients. Of the 122 patients with neurological etiology of SM, 51 (41.8%) had a known past neurological history, and of the 30 patients with psychiatric etiology, only seven (23.3%) were known to have a past psychiatric history.

The time elapsed between patient arrival at the ED and the activation of the stroke code varied significantly between the stroke and stroke-mimic groups. Stroke patients had a shorter time from arrival to activation (median of 7.5 [IQR 8.5 minutes]) compared to SM patients (median of 11 [IQR 15 minutes], *P* < .001). Almost all patients (99.3%) who presented to the ED received brain imaging during the visit.

Stroke-mimic patients were less likely to have computed tomography angiography (CTA) (42.1% vs 57%; *P* < .001) and magnetic resonance angiography (MRA) (20.4% vs 39.3%; *P* < .001). Thirty-three patients (5.7%) received thrombolysis with recombinant tPA, one of whom had a stroke mimic (Table 2). Table 3 shows the patients’ vital signs and symptoms upon ED presentation. Stroke patients presented more with speech abnormalities (64.8% vs. 49.4; *P*< .001), extremities weakness (63% vs 29.8%; *P* < .001), or facial weakness (30.7% vs. 19.1%; *P* = .002). They were also found to have significantly higher mean systolic (151.8 ± 26.3 vs 144.0 ± 27.4; *P* = .001).

## DISCUSSION

To the best of our knowledge, this study is the first to describe the rate and characteristics of stroke mimics in Lebanon. Roughly half of our patients (40.2%) were found to have SM. Roughly half of our patients (40.2%) were found to have SM. This incidence of mimics is more than double the reported global average of 22%^4^ and is higher than the reported figures in regional studies from Qatar (35%)^22^ and Morocco (15.6%).^23^ Conversely, our findings are similar to those reported in a study from Canada (43.2%).^24^ With no prior data on the incidence of SMs in other local hospitals, it is difficult to pinpoint the origin of our high rate and to comment on the sensitivity of our stroke code-activation practices. Several studies have proposed an algorithm for the early detection of a mimic^25^,^26^; however, in the absence of a standardized and validated mimic identification model, the activation of the stroke code must rely on the judgment of the triage team and the internal protocol set by each medical care facility. Of the 235 SM patients, 122 (51.9%) had a non-cerebrovascular neurologic origin. Seizures, as a single disease diagnosis, were the most common presentation for SMs. This mirrors globally reported trends, which state that seizures cause most SM cases.^3^,^6^ Nevertheless, global incidence accounts for an average of 20% of said cases^5^,^10^ as compared to 14.0% in our study. The lower number of seizure disorders presenting as a stroke to our ED could be due to an early identification of neurologic comorbidities at the triage level, prompting the treatment of the patient as a regular neurologic case rather than a potential stroke case. A patient presenting in the postictal phase could be accompanied by a chaperone who witnessed the seizure, which decreases the likelihood of a stroke diagnosis.

Our data revealed that 30 patients (12.8%) had functional SM. This incidence was similar to that found in the United Kingdom (13%)^27^ and Canada (11.9%),^28^ lower than that found in France (16.7%)^29^ and Saudi Arabia (24.4%)7, and higher than that found in Korea (5.6%)10 and Iran (8.1%).^30^ Cultural distinctions and variations in illness manifestation behaviors across countries could account for these differences. This is substantiated by data from Qatar initially showing a 17% incidence of functional SM.^31^ However, when including all nationalities presenting to the same center, this incidence increased to 29.2%.^31^ Interestingly, our incidence paralleled that from SM studies in other EDs (11.9%, 8.1%, 5.6%)^10^,^28^,^30^ as opposed to studies conducted in stroke centers where psychiatric disorders were identified as one of the highest proportions of all causes of mimics (29.2%, 25.7%, 24.4%).^7^,^23^,^31^ This discrepancy may be explained by the fact that the primary assessors in the former studies were neurologists, while those in the latter studies, including ours, were emergency physicians. The more diversified exposure to psychiatric cases during emergency medicine training may be related to the lesser percentage observed. It may also be skewed by self-selection bias, whereby functional patients, exhibiting stroke symptoms, would be more likely to present to a stroke center rather than to an ED.

Several clinical features were found to be associated with SM in our sample. Age <65 years was independently associated with SM. This is in line with the published literature on younger individuals being more likely to have SM.^10^,^12^–^14^ This is also expected, considering that 75% of all strokes occur in persons ≥65 years of age.^11^ Past medical history and presenting symptoms were also consistent with prior studies in terms of risk factors. A history of hypertension or atrial fibrillation, or motor deficits such as extremity weakness or speech disturbances favored a TS diagnosis.^9^,^10^,^15^,^32^,^33^

In contrast, a history of psychiatric or neurological disease or an altered mental status presentation increased the odds of a SM diagnosis.^10^,^15^ Specifically, in our dataset, a history of neurological diseases other than stroke strongly favored SM diagnoses. This can be explained by the fact that individuals with a history of seizures can present in a postictal state, mimicking strokes.^15^,^34^ Seizures are one of the most common SM presentations in both our data and in the literature.^34^,^35^ Additionally, previously published studies have shown that migraines, especially those with aura or hemiplegic migraines, are one of the leading SM presentations, and having a history of migraines increases the likelihood of an SM diagnosis compared to a TS. Furthermore, due to increased intracranial pressure or mass effect, patients with brain lesions can present with neurological deficits like those seen in strokes, despite the absence of any vascular event.^5^,^36^

The results obtained in our study suggest the need for a more specific algorithm for activating a stroke code. This could include the involvement of more physicians and personnel with exposure to neurologic and psychiatric training in the triage of suspected stroke cases and the subsequent activation of the stroke code. Our data also highlight the need for more studies on the incidence of SMs in various medical care facilities across Lebanon. A local average stroke-mimic rate would allow for a better evaluation of the specificity and efficiency of the stroke code system in hospitals nationwide.

## LIMITATIONS

The study is limited to patients presenting to the ED of a single, tertiary-care center in Lebanon. This limits the generalizability of the results we reported and may not reflect the true rate of SMs in the country. The lack of data from other local hospitals makes it difficult to compare our results to other care centers, thus diminishing the ability to accurately describe the incidence of SMs as well as the sensitivity of our stroke code-activation system. In addition, the total number of patients treated with r-tPA was low, making it difficult to truly evaluate the negative outcomes of misidentifying and wrongly treating SM cases.[Fig f2-wjem-26-943]

Moreover, this study has methodological limitations common to retrospective reviews. The data were examined retrospectively, and the diagnoses at discharge were extracted as ICD-10-CM codes from the charts of identified patients. The diagnoses may have been classified and labeled incorrectly, making some etiologies over- or under-represented. Also, not all Worster et al criteria for retrospective chart reviews were followed.^21^ Specifically, the performance of data abstractors was not formally monitored, they were not blinded to the study objectives, and data extraction was conducted once; thus, interobserver reliability was not formally tested or reported, which may have introduced bias. However, to reduce this risk, we used a structured data abstraction form with predefined variables to ensure consistency. Additionally, all data abstractors were medically trained and had extensive prior experience with the database, which contributed to the consistency and accuracy of data entry.

## CONCLUSION

Given that stroke is a time-dependent medical emergency, it is imperative that emergency physicians rule out acute stroke first. However, given that nearly half of the presenting cases received a diagnosis of stroke mimic, it is imperative to devise a more specific screening pathway for stroke code activation. In low- and middle-income countries, specifically Lebanon, unwarranted code activations overburden the fragile healthcare system, which already suffers from limited capacities characterized by insufficient staff, equipment, and medications. Thus, a step forward toward developing a psychometrically robust and culturally adapted assessment for stroke mimics may help physicians in triaging suspected patients and considering alternative diagnoses.

## Supplementary Information





## Figures and Tables

**Figure 2 f2-wjem-26-943:**
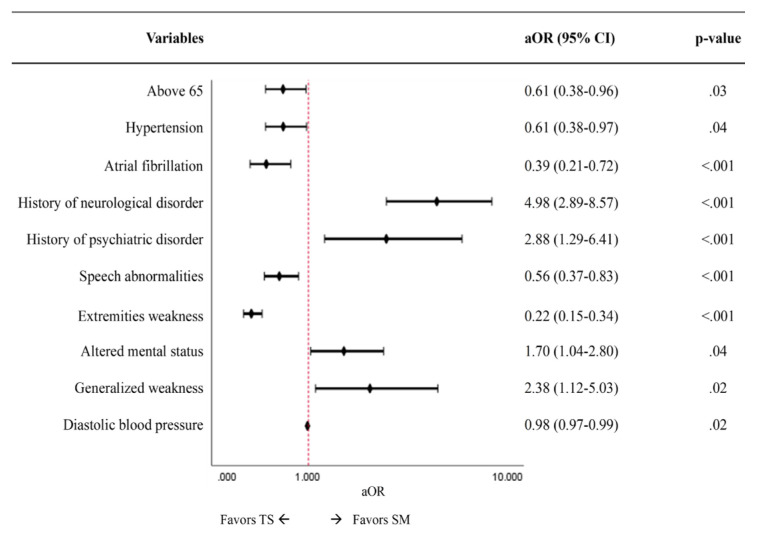
Multivariate logistic regression analysis identifying factors associated with stroke mimic vs. true stroke among patients with stroke code activation. *aOR*, adjusted odds ratio; *CI*, confidence interval; *TS*, true stroke; *SM*, stoke mimic. *ED*, emergency department; *TS*, true stroke; *SM*, stroke mimic; *mmHg*, millimeters of mercury.

**Table 1:** Adherence to recommended retrospective chart review methodology based on Worster et al criteria.

Method criterion	Details
Abstractor training	Data abstractors had prior training in using the EHR for clinical purposes.
Case selection criteria	We defined inclusion of cases as patients who had stroke code activation upon presentation to the ED. We excluded patients with incomplete service at our facility.
Variable definition	We defined and agreed on all variables before data extraction.
Abstraction forms	A standardized data extraction form was prepared as part of study conceptualization and approved by the IRB.
Medical record identified	The database used in our study was the EHR.
Sampling method	We reviewed all patients who had stroke code activation, identified by administrative record.
Missing-data management plan	Missing data is reported in the study as “unknown.”
Institutional review board approval	The study was approved by the IRB.

*EHR*, electronic health record; *ED*, emergency department; *IRB*, institutional review board.

**Table 2:** Demographics and emergency department visit characteristics of patients presenting with stroke activation: stroke mimics vs, true stroke patients.

		TS (N=349)	SM (N=235)	*P*-value
Patient demographics				
Age (years, mean ± SD)		70.6 ± 13.5	64.7 ± 16.0	<.001
>65 years		234 (67.0%)	120 (51.1%)	<.001
Sex	Male	200 (57.3%)	118 (50.2%)	.09
	Female	149 (42.7%)	117 (49.8%)	
Marital status	Single	47 (13.5%)	29 (12.3%)	.80
	Married	281 (80.5%)	189 (80.4%)	
	Widowed or divorced	21 (6%)	17 (7.2%)	
Smoking status	Never	143 (41.0%)	96 (40.9%)	.38
	Current	101 (28.9%)	76 (32.3%)	
	Former	92 (26.4%)	50 (21.3%)	
	Unknown	13 (3.7%)	13 (5.5%)	
Past medical history	Hypertension	253 (72.5%)	144 (61.3%)	< .01
	Diabetes mellitus	118 (33.8%)	77 (32.8%)	.79
	Dyslipidemia	163 (46.7%)	98 (41.7%)	.23
	Coronary artery disease	111 (31.8%)	44 (18.7%)	.001
	Atrial fibrillation	82 (23.5%)	20 (8.5%)	<.001
	Carotid artery disease	4 (1.1%)	3 (1.3%)	1
	Transient ischemic attack	29 (8.3%)	17 (7.2%)	.64
	Ischemic stroke	73 (21.0%)	25 (10.6%)	.001
	Hemorrhagic stroke	6 (1.7%)	5 (2.1%)	.76
	Malignancy	42 (12.0%)	36 (15.3%)	.25
	Psychiatric disorder	15 (4.3%)	27 (11.5%)	.001
	Other neurological disorders*	34 (9.7%)	70 (29.8%)	<.001
ED visit characteristics				
Time arrival to activation (min)		7 (8.5)	11 (15)	<.001
CT with and without contrast		227 (65%)	165 (70.2%)	.19
CT angiography		199 (57%)	99 (42.1%)	<.001
MRI with and without gadolinium		136 (39.0%)	89 (37.9%)	.80
MR angiography		137 (39.3%)	48 (20.4%)	<.001
r-tPA		32 (9.2%)	1 (0.4%)	<.001
ED disposition	Home	11 (3.2%)	37 (15.7%)	<.001
	Admit	338 (96.8%)	195 (83%)	
	Dead	0 (0%)	3 (1.3%)	

* Previous history of neurological disorders other than stroke or transient ischemic attack (including seizures, migraines, Alzheimer’s disease, brain lesions, etc).

*TS*, true stroke; *SM*, stroke mimic; *CT*, computed topography; *MRI*, magnetic resonance imaging; *r-tPA*, recombinant tissue plasminogen activator; *ED*, emergency department.

**Table 3:** Symptoms and vital signs at ED presentation of stroke-mimic and true-stroke patients with stroke code activation.

	TS (N=349)	SM (N=235)	P-value
Presenting symptoms			
Speech abnormalities	226 (64.8%)	116 (49.4%)	<.001
Visual abnormalities	17 (4.9%)	13 (5.5%)	.72
Extremity weakness	220 (63%)	70 (29.8%)	<.001
Extremity numbness	57 (16.3%)	46 (19.6%)	.31
Facial weakness	107 (30.7%)	45 (19.1%)	< .01
Facial numbness	22 (6.3%)	27 (11.5%)	.03
Headache	34 (9.7%)	44 (18.7%)	< .01
Altered mental status	58 (16.6%)	71 (30.2%)	<.001
Loss of consciousness	19 (5.4%)	23 (9.8%)	.05
Generalized weakness	18 (5.2%)	28 (11.9%)	< .01
Triage Vital Signs			
Temperature (°C)	36.7 ± 0.3	36.8 ± 0.6	.15
Pulse (beats per minute)	83.0 ± 19.4	86.4 ± 18.8	.03
Systolic blood pressure (mmHg)	151.8 ± 26.3	144.0 ± 27.4	.001
Diastolic blood pressure (mmHg)	85.6 ± 19.0	82.5 ± 15.8	.03
Oxygen saturation (%)	97.6 ± 3.8	97.4 ± 4.4	.55
Respiratory rate (breaths per minute)	18.4 ± 3.5	19.2 ± 5.2	.04
