# Supplementary figures and images for: The Incidence of Stroke Mimics in the Emergency Department of a Tertiary-care Center in Lebanon
Source: West J Emerg Med. 2025 Jul 18;26(4):943–50. doi: 10.5811/westjem.39718 (PMC12342410; doi:10.5811/westjem.39718)

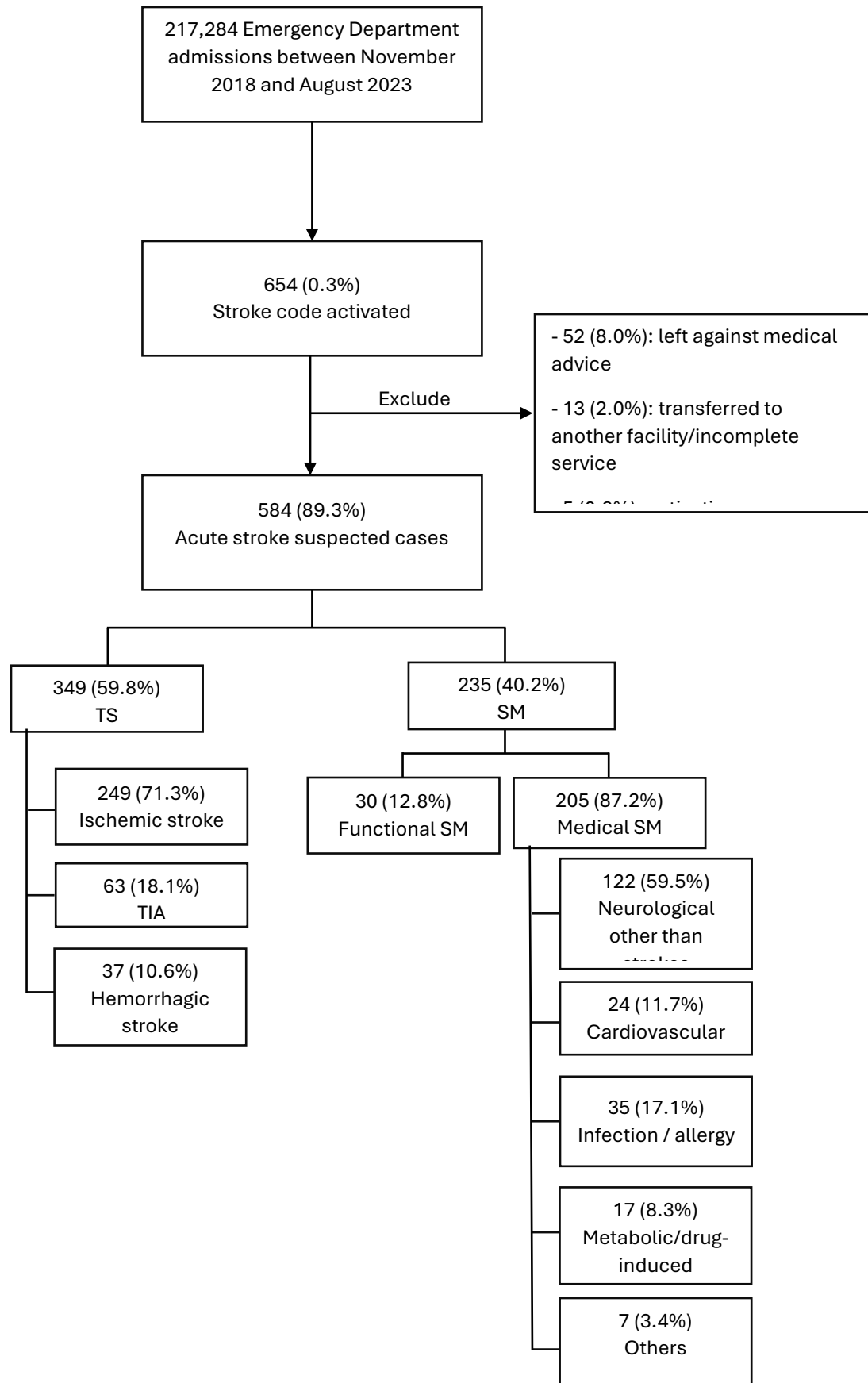

Supplement: Supplementary file 1 [file wjem-26-943-g001.pdf]

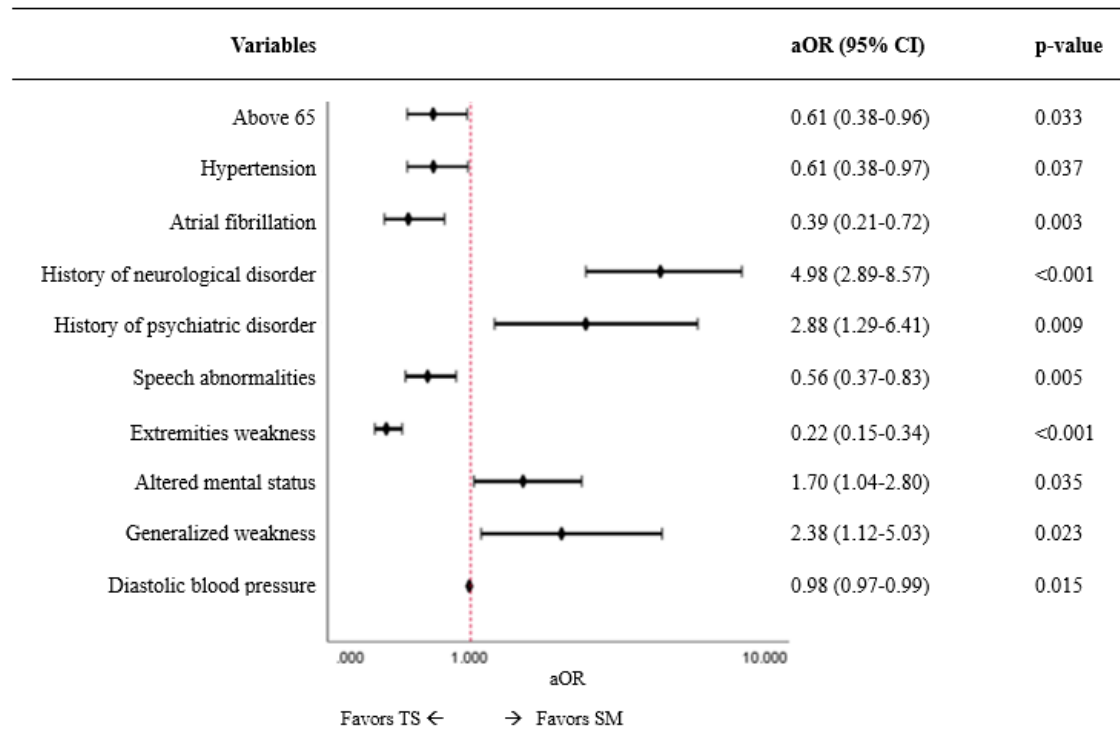

Supplement: Supplementary file 2 [file wjem-26-943-g002.pdf]
